# Gut microbiota mediates the anti-obesity effects of *Gnaphalium affine* methanol extract in HFD-induced obesity

**DOI:** 10.3389/fnut.2026.1779459

**Published:** 2026-03-13

**Authors:** Zhihao Yu, Huaizhi Ma, Huiyuan Zhang, Linlin Gao, Xinrui Fu, Haoyu Wang, Yao Xiao, Xiaojuan Wu, Andong Zhang, Yingqian Kang, Guzhen Cui, Zhenghong Chen, Daoyan Wu

**Affiliations:** 1 Key Laboratory of Microbio and Infectious Disease Prevention and Control in Guizhou Province, Key Laboratory of Microbiology and Parasitology of Education Department of Guizhou, School of Basic Medical Science, Guizhou Medical University, Guiyang, China; 2 Department of Emergency Medicine Center, Sichuan Provincial People’s Hospital, University of Electronic Science and Technology of China, Chengdu, Sichuan, China

**Keywords:** functional food, *Gnaphalium affine*, gut microbiota, metabonomics, obesity

## Abstract

**Background:**

Obesity constitutes a pressing global public health challenge, characterized by intricate associations with metabolic dysregulation and gut microbiota dysbiosis.

**Methods:**

This study systematically evaluated the anti-obesity efficacy and underlying mechanisms of *Gnaphalium affine* methanol extract (GAE) in high-fat diet (HFD)-induced obese mice, with integrated fecal microbiota transplantation (FMT) experiments to establish causal relationships between GAE-modulated gut microbiota and metabolic improvements.

**Results:**

GAE intervention significantly ameliorated HFD-induced metabolic disorders, as evidenced by reduced oxidative stress, enhanced glucose tolerance, suppressed visceral adiposity, and attenuated chronic low-grade inflammation. Mechanistically, GAE preserved intestinal barrier integrity through upregulation of tight junction protein expression. Multi-omics integration of 16S rRNA gene sequencing and untargeted metabolomics revealed that GAE substantially rectified gut microbiota dysbiosis and lipid metabolic disturbances, mediated by specific bioactive metabolites—including lysophosphatidylcholine 22:6 and N-oleoyl glycine—and enrichment of beneficial bacterial genera (*Phascolarctobacterium* and *Lactobacillus*). Critically, FMT experiments demonstrated that the gut microbiota remodeled by GAE administration was sufficient to transfer obesity-alleviating phenotypes to recipient mice.

**Conclusion:**

Collectively, these findings establish that GAE exerts multi-target anti-obesity effects through modulation of the “microbiota–gut–metabolism” axis, providing compelling preclinical evidence supporting the development of GAE as a functional food ingredient for weight management applications.

## 1 Introduction

Obesity is one of the leading risk factors for chronic disease and death worldwide. As a complex condition, obesity exerts systemic pathophysiological effects through multiple interconnected mechanisms, including chronic low-grade inflammation, metabolic dysregulation, hormonal imbalances, oxidative stress, adipose tissue dysfunction and perturbations in gut microbiota (1). These mechanisms collectively drive the development of a variety of chronic diseases, including cardiovascular disease, diabetes, liver disease, and certain types of cancer (2). Increasing evidence indicates that intestinal microbiota dysbiosis can disrupt host metabolism and immune function, contributing to obesity and metabolic diseases (3). Research has indicated that Gut microbiota enhances host energy extraction/storage via gene expression changes, contributing to weight gain (4). Moreover, dysbiosis disrupts the intestinal barrier integrity, leading to elevated systemic levels of lipopolysaccharides (LPS). This “metabolic endotoxemia” triggers chronic low-grade inflammation via Toll-like receptor 4 (TLR4) signaling, which is strongly associated with the pathogenesis of insulin resistance and atherosclerosis (5–7). Consequently, targeting the gut microbiota through dietary interventions, such as prebiotics and functional foods, has become a promising therapeutic strategy to alleviate obesity and its metabolic complications (8).

*Gnaphalium affine*, a traditional medicinal and edible plant known as Qingming grass or Buddha’s ear grass, is extensively consumed as a wild vegetable in China. Previous studies have demonstrated that *Gnaphalium affine* exhibits a range of health-promoting properties, including anti-inflammatory, antioxidant, and gut microbiota-modulating effects (9–13). As a traditional Miao medicinal herb, it is clinically employed to promote lung dispersion and alleviate asthma, eliminating phlegm and stopping cough, tonifying spleen, heat-clearing and detoxifying, strengthening the middle warmer and benefiting vital energy. These pharmacological effects are linked to the attenuation of obesity-associated inflammation and oxidative stress. Phytochemical analyses have revealed that *Gnaphalium affine* is rich in bioactive compounds such as flavonoids (e.g., quercetin, kaempferol, luteolin), phenolic acids (e.g., chlorogenic acid, caffeic acid), and polysaccharides (14–16). These components have been individually reported to improve lipid metabolism and modulate gut microbiota composition (17–21). However, despite its potential, the specific effects of *Gnaphalium affine* methanol extract (GAE) on high-fat diet (HFD)-induced obesity and whether these effects are mediated by the gut microbiota remain largely unexplored.

To bridge this gap, our study systematically investigated the anti-obesity effects of GAE and its underlying mechanisms. We hypothesized that GAE alleviates HFD-induced metabolic disorders by modulating the gut microbiota. To test this, we integrated a comprehensive suite of analyses, including multi-omics profiling (16S rRNA sequencing and untargeted metabolomics) and, crucially, a fecal microbiota transplantation (FMT) experiment to establish a causal link. Our findings provide a strong scientific basis for the application of *Gnaphalium affine* in functional foods for metabolic health.

## 2 Materials and methods

### 2.1 Detection of active substances in GAE

Twenty grams of GA powder was homogenously mixed with methanol and prepared the crude extract according our previous method with modification (22). This extract of GA (GAE) was used an ultra-high performance liquid chromatography-tandem mass-spectrometry (LC-MS/MS, Thermo Fisher, Germany) analysis.

### 2.2 Animal experiments

Male C57BL6/J mice, aged 6 weeks, were purchased from the Laboratory Animal Engineering Technology Center of Guizhou Medical University (Guizhou, China). Male mice were used to avoid the confounding effects of the estrous cycle on metabolism and gut microbiota. The Animal Care Committee guidelines were adhered to for animal care and experimental procedures, with approval from the Animal Experimental Ethical Inspection of Guizhou Medical University (ethics approval number: 2100089). During acclimatization, food and water were provided ad libitum. Following acclimatization, the mice were randomly assigned to four groups (*n* = 5 per group): (1) Group NC (normal chow); (2) Group NCGP (normal chow plus intragastric administration by 80 mg/kg GAE); (3) Group HFD (high-fat diet); and (4) Group HFDGP (high-fat diet plus intragastric administration of 80 mg/kg GAE). In addition, to prevent coprophagia and social stress, all mice were housed individually in specific pathogen-free (SPF) cages under controlled environmental conditions (22 ± 2°C, 12-h light/dark cycle). All interventions lasted for 12 weeks. Body weight was measured weekly. Fecal samples were collected daily from all four groups during the first 4 weeks for fecal microbiota transplantation (FMT) and subsequent analyses. Fecal samples were preserved at -80°C for future analysis. At the beginning of week 12, a glucose tolerance test (GTT) was performed. After a 5-day recovery period, an insulin tolerance test (ITT) was conducted. Finally, following a 12-h fast the next day, all mice were euthanized by cervical dislocation under deep anesthesia induced by isoflurane inhalation (3% in oxygen). Blood samples were obtained by centrifuging the blood at 300 rpm for 20 min to obtain serum, which was then stored at -80°C. Intestine, liver, and epididymal white adipose tissue (eWAT) were stored at -80°C. Sections of the intestine and epididymal fat tissue were fixed in 4% paraformaldehyde.

### 2.3 Fecal microbiota transplantation

Prior to FMT, recipient mice underwent a 1-week acclimatization period as previously described. Mice were administered a combination of ampicillin (1 g/L), metronidazole (1 g/L), vancomycin (0.5 g/L), ciprofloxacin (0.2 g/L), and neomycin (0.5 g/L) in their drinking water for 1 week to induce a pseudo germ-free state. Subsequently, the mice continued on a high-fat diet (HFD) for the duration of the FMT experiment. For FMT, fecal samples collected daily from each donor mouse group starting the fourth week of the feeding experiment were homogenized in saline with 0.1% cysteine (200 mg/2 mL) and centrifuged at 1000 g for 5 min. Recipient mice were intragastrically administered 100 μL of the respective bacterial suspension and assigned to Group FNC, FNCGP, FHFD, and FHFDGP; each group consisted of five mice randomly assigned. Starting from the fourth week, the FMT was conducted daily. Mice body weights were recorded on a weekly basis. Fecal samples from each group of recipient mice were collected daily and stored at -80°C for subsequent analysis. Blood and tissues were collected post-sacrifice as previously described.

### 2.4 Glucose and insulin tolerance test

At the beginning of week 12, a Glucose Tolerance Test (GTT) was performed. The mice were fasted overnight for 12 h at the conclusion of the feeding protocol. During the fasting period, only regular water was provided. In GTT experiment, mice received an intraperitoneal injection of a glucose solution (2 g/kg body weight) using a syringe with a feeding needle. Blood glucose levels were measured from tail vein blood at 0, 30, 60, 90, and 120 min using an ACCU-CHEK Performa glucometer (Roche). After a 5-day recovery period, an Insulin Tolerance Test (ITT) was conducted in mice that had been fasted for 4–6 h. Insulin (0.75 U/kg body weight) was injected intraperitoneally, and blood glucose was measured at the same time points as in the GTT.

### 2.5 Biochemical and immunological assays

Serum concentrations of triglycerides (TG), total cholesterol (TC), low-density lipoprotein (LDL), high-density lipoprotein (HDL), malondialdehyde (MDA),, as well as the activities of superoxide dismutase (SOD), glutathione peroxidase (GSH-Px), aspartate aminotransferase (AST) and alanine aminotransferase (ALT), were quantified using commercially available assay kits (Shanghai Enzyme-linked Biotechnology Co., Ltd., Shanghai, China). Levels of IL-6, IL-1β, MCP-1, and insulin were determined by enzyme-linked immunosorbent assay (ELISA) kits (Nanjing Byabscience Biotechnology Co., Ltd., Nanjing, China).

### 2.6 Hematoxylin and Eosin (HE) stain and immunohistochemistry (IHC)

HE stain and IHC was performed according to standard histological protocols. In the IHC experiment, primary antibodies p65, Occludin, Claudin-1, and ZO-1, along with an HRP-conjugated secondary antibody, were used as per the provided instructions. All antibodies were purchased from WanleiBio Co., Ltd. (Dalian, China).

### 2.7 The joint analysis of 16S rRNA sequencing and untargeted metabolomics

Fresh fecal samples were collected and immediately stored at −80°C. Total genomic DNA was extracted using the CTAB method, and the V3-V4 hypervariable region of the 16S rRNA gene was sequenced on an Illumina MiSeq PE250 platform by Wekemo Tech Group Co., Ltd. (Shenzhen, China). Raw reads were quality-filtered, denoised, and chimera-removed using QIIME2 (v2022.2) with the DADA2 plugin to generate Amplicon Sequence Variants (ASVs). Taxonomy was assigned against the SILVA database (release 138) with a confidence threshold of 0.7. Alpha and Beta diversity analyses were performed using QIIME2, and differential taxa were identified using LEfSe analysis (LDA score > 3.0, *p* < 0.05). For untargeted metabolomics, fecal samples were extracted with pre-chilled 80% methanol, sonicated, and centrifuged. The supernatant was freeze-dried, reconstituted in 10% methanol, and analyzed using a Vanquish UHPLC system coupled with an Orbitrap Q Exactive™ HF mass spectrometer (Thermo Fisher). Raw data were processed using Compound Discoverer 3.1 (Thermo Fisher) for peak alignment, picking, and normalization to total spectral intensity. Metabolite identification was performed using the mzCloud, mzVault, and ChemSpider databases. Differential metabolites were identified based on Variable Importance in Projection scores > 1.0 (from OPLS-DA) and *p* < 0.05. Integrative analysis of 16S rRNA and metabolomics data was conducted on the Wekemo Bioincloud platform^1^. Microbiome data were Hellinger-transformed, and correlations with metabolites were assessed using Spearman’s rank correlation (|r| > 0.2, *p* < 0.05). All sequencing data are available in the NCBI Sequence Read Archive (SRA) under accession number PRJNA947507.

### 2.8 Statistical analysis

Statistical analyses were conducted using GraphPad Prism software (Version 8.02). Statistical analyses were conducted using the Student’s *t*-test, one-way ANOVA, or two-way ANOVA. Correlation was determined by Pearson’s correlation. *p* < 0.05 was deemed statistically significant.

## 3 Results

### 3.1 The components and antioxidant activity of GAE

Comprehensive metabolite profiling of GAE was performed using LC-MS analysis, with 455 distinct compounds identified and quantified in the positive ion mode, while 255 unique metabolites were detected in the negative ion mode. By querying the biological activities of relevant components, we screened out 28 compounds that are related to lipid metabolism or have antioxidant functions. Specific details are shown in Table 1.

**TABLE 1:** The main components related to lipid metabolism and antioxidation in GAE.

NO.	Ingredient	Formula	Molecular weight	RT (min)	Scan mode
1	Carnosic acid	C_20_H_28_O_4_	332.1984	5.866	+
2	Scopoletin	C_10_H_8_O_4_	192.0423	5.52	+
3	Choline	C_5_H_13_NO	103.0996	1.569	+
4	DL-Carnitine	C_7_H_15_NO_3_	161.1051	1.599	+
5	Nicotinic acid	C_6_H_5_NO_2_	123.032	2.031	+
6	Pantothenic acid	C_9_H_17_NO_5_	219.1106	5.057	+
7	Alpha-Linolenic acid	C_18_H_30_O_2_	278.2245	9.716	+
8	Myricetin	C_15_H_10_O_8_	318.0379	5.442	+
9	Luteolin	C_15_H_10_O_6_	286.0476	5.868	+
10	Apigenin	C_15_H_10_O_5_	270.0526	6.067	+
11	Coumarin	C_9_H_6_O_2_	146.0367	2.397	+
12	Ascorbyl palmitate	C_22_H_38_O_7_	431.2884	5.877	+
13	Trigonelline	C_7_H_7_NO_2_	137.0477	5.484	+
14	Citric acid	C_6_H_8_O_7_	146.0214	2.341	–
15	Fumaric acid	C_4_H_4_O_4_	116.0107	1.686	–
16	Quercetin	C_15_H_10_O_7_	302.0425	5.8	–
17	Chlorogenic acid	C_16_H_18_O_9_	354.0951	5.417	–
18	Thioctic acid	C_8_H_14_O_2_S_2_	206.0425	5.396	–
19	Gallic acid	C_7_H_6_O_5_	170.0213	2.894	–
20	Caffeic acid	C_9_H_8_O_4_	134.0365	5.192	–
21	Hydroxytyrosol	C_8_H_10_O_3_	154.0628	5.355	–
22	Eriodictyol	C_15_H_12_O_6_	288.0634	5.74	–
23	Genistein	C_15_H_10_O_5_	270.0528	7.917	–
24	Abscisic acid	C_15_H_20_O_4_	264.1362	6.271	–
25	Esculetin	C_9_H_6_O_4_	178.0262	5.341	–
26	Protocatechuic acid	C_7_H_6_O_4_	154.0264	5.029	–
27	Catechol	C_6_H_6_O_2_	110.0366	5.03	–
28	Syringic acid	C_9_H_10_O_5_	198.0524	4.911	–

GAE, *Gnaphalium affine* methanol extract.

**FIGURE 1:**
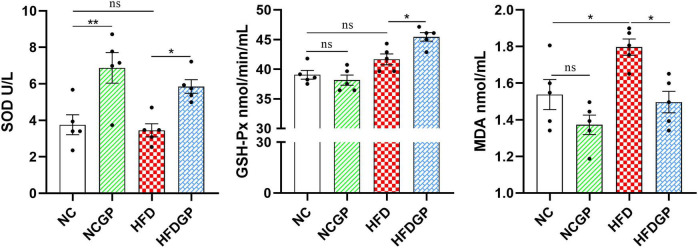
The antioxidant activity of GAE (*N* = 5). NC, normal chow mice; NCGP, normal chow mice plus GAE; HFD, high-fat diet mice; HFDGP, high-fat diet mice plus GAE. GAE, *Gnaphalium affine* methanol extract; SOD, superoxide dismutase; GSH-Px, glutathione peroxidase; MDA, malondialdehyde. *P < 0.05; **P < 0.01; and ns *P* > 0.05.

As shown in Figure 1, the SOD level in Group NCGP was significantly higher than that in Group NC, indicating that under normal dietary conditions, consuming GAE can enhance SOD activity in mice. Meanwhile, the level of SOD and GSH-Px in Group HFDGP was significantly higher than that in Group HFD, suggesting that consuming GAE can improve the reduction of SOD and GSH-Px activity caused by a high-fat diet. Finally, the MDA content in Group NC, NCGP group, HFD group and HFDGP group was 1.54, 1.37, 1.80, and 1.50 nmol/mL, respectively, revealing that the administration of GAE could alleviate the oxidative stress level in mice.

### 3.2 GAE attenuates high-fat diet induced obesity

**FIGURE 2:**
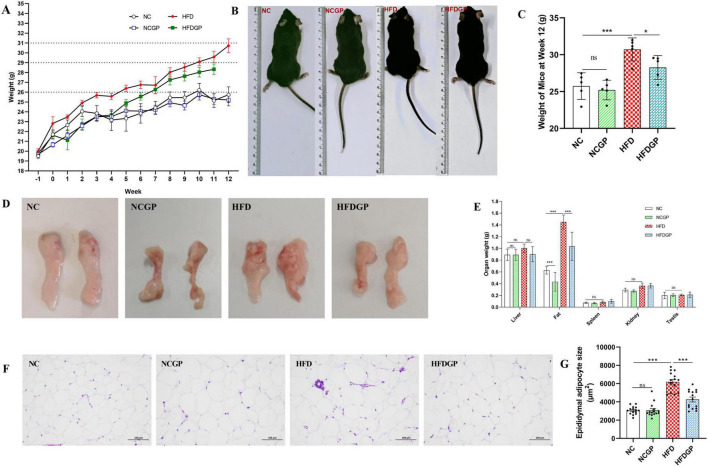
The effects of GAE on the body weight and epididymal fat (*N* = 5). **(A)** body weight of mice; **(B)** body size of mice; **(C)** body weight of mice in 12th week; **(D)** morphological observations of epididymal adipose tissue; **(E)** weights of different organs; **(F)** HE staining of epididymal adipose; **(G)** mean adipocyte size of epididymal adipose tissue sections. NC: normal chow mice; NCGP: normal chow mice plus GAE; HFD: high-fat diet mice; HFDGP: high-fat diet mice plus GAE. GAE: *Gnaphalium affine* methanol extract. *P < 0.05; ***P < 0.001; and ns *P* > 0.05.

Changes in body weight of GAE-treated experimental mice are shown in Figure 2A. Group NC exhibited a relatively stable weight gain pattern. Mice fed a high-fat diet (HFD group) demonstrated significantly greater weight gain compared to Group NC, indicating successful induction of weight gain through the high-fat dietary regimen. In contrast, Group HFDGP displayed a comparatively slower rate of weight gain, suggesting that gallic acid equivalent (GAE) may potentially inhibit body weight accumulation. At week 12, the body weight of mice in Group HFDGP was significantly lower than that in Group HFD (Figures 2B,C). As shown in 2E, the weights of the liver, spleen, kidney, and testes showed no significant differences among the groups. Compared with Group HFD, the epididymal fat weight in Group HFDGP was significantly reduced (Figures 2D,E). As shown in Figures 2F,G, the HE staining results demonstrated that adipocytes in Group HFD were significantly larger than those in Group NC, with plump cellular morphology. In contrast, Group HFDGP exhibited a marked reduction in adipocyte size compared than Group HFD (*P* < 0.001), accompanied by a more regular cellular morphology.

**FIGURE 3:**
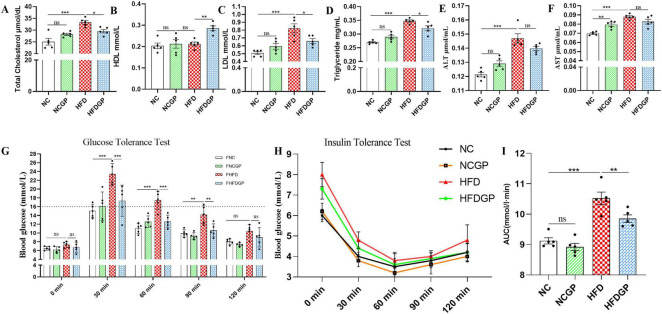
Effect of GAE on obesity-related biochemical indicators in mice (*N* = 5). **(A)** Serum total cholesterol levels; **(B)** Serum high-density lipoprotein cholesterol (HDL-C) levels; **(C)** the low-density lipoprotein cholesterol (LDL-C) levels in serum; **(D)** the triglyceride levels in serum; **(E)** the alanine aminotransferase (ALT) levels in serum; **(F)** the aspartate aminotransferase (AST) levels in serum; **(G)** blood glucose levels during the glucose tolerance test (GTT); **(H)** blood glucose levels during the insulin tolerance test (ITT); **(I)** the quantitative analysis of the area under the curve (AUC) of the insulin tolerance test. NC, normal chow mice; NCGP, normal chow mice plus GAE; HFD, high-fat diet mice; HFDGP, high-fat diet mice plus GAE. GAE, *Gnaphalium affine* methanol extract; HDL, high-density lipoprotein; LDL, low-density lipoprotein; ALT, alanine aminotransferase; AST, aspartate aminotransferase. *P < 0.05; **P < 0.01; ***P < 0.001; and ns *P* > 0.05.

**FIGURE 4:**
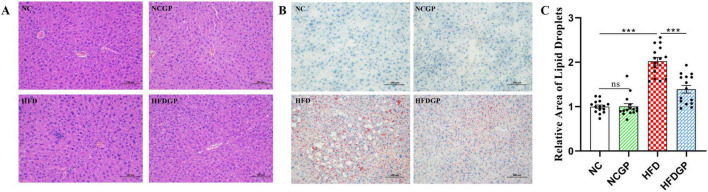
HE staining and Oil Red staining of livers in mice (*N* = 5). **(A)** HE staining of liver; **(B)** Oil Red staining of liver; **(C)** quantitative of lipid droplet area of Oil Red staining. NC: normal chow mice; NCGP: normal chow mice plus GAE; HFD: high-fat diet mice; HFDGP: high-fat diet mice plus GAE. ***P < 0.001; and ns *P* > 0.05.

High-fat diet feeding significantly increased total cholesterol levels, whereas supplementation with GAE in HFD-fed mice markedly attenuated this elevation (Figure 3A). Compared with Group HFD, Group HFDGP demonstrated increased high-density lipoprotein (HDL) levels and significantly reduced low-density lipoprotein (LDL) concentrations (Figures 3B,C). Triglyceride concentrations in the NC, NGCP, HFD, and HFDGP groups were 0.27, 0.29, 0.35, and 0.32 mg/mL, respectively, with Group HFDGP showing significantly lower levels than Group HFD (Figure 3D). AS shown in Figures 3E, F, the levels of alanine aminotransferase (ALT) and aspartate aminotransferase (AST) in Group HFD were significantly higher than those in Group NC. The ALT level in Group HFDGP was significantly lower than that in Group HFD. This indicates that GAE has a certain protective effect on liver function in mice fed a high-fat diet. As demonstrated in Figure 3G, Group HFD showed significantly elevated blood glucose levels compared to both NC and HFDGP groups at 30, 60, and 90 min post-glucose administration. Notably, no intergroup differences in blood glucose levels were observed at 120 min. ITT assay revealed that, compared with Group NC and NCGP, mice fed a high-fat diet exhibited significantly impaired insulin sensitivity (Figures 3H,I). As can be seen from Figure 4A, the morphology of hepatocytes in Group NCGP was similar to that in Group NC, without obvious abnormalities observed. Hepatocytes in Group HFD showed obvious steatosis, cell swelling, and a small amount of inflammatory cell infiltration, indicating that a high-fat diet could cause pathological changes in the liver. The degree of steatosis in hepatocytes in Group HFDGP was less than that in Group HFD. At the same time, quantitative analysis of Oil Red staining sections revealed significantly less lipid accumulation in HFDGP group livers compared to HFD group (Figures 4B,C).

In conclusion, GAE supplementation significantly attenuated high-fat diet-induced weight gain and adipocyte hypertrophy in mice, concurrently ameliorating dyslipidemia while demonstrating hepatoprotective effects through improved ALT/AST levels, restored glucose homeostasis, and reduced lipid accumulation in hepatocytes.

### 3.3 GAE attenuates HFD-induced systemic inflammation and intestinal barrier dysfunction

**FIGURE 5:**
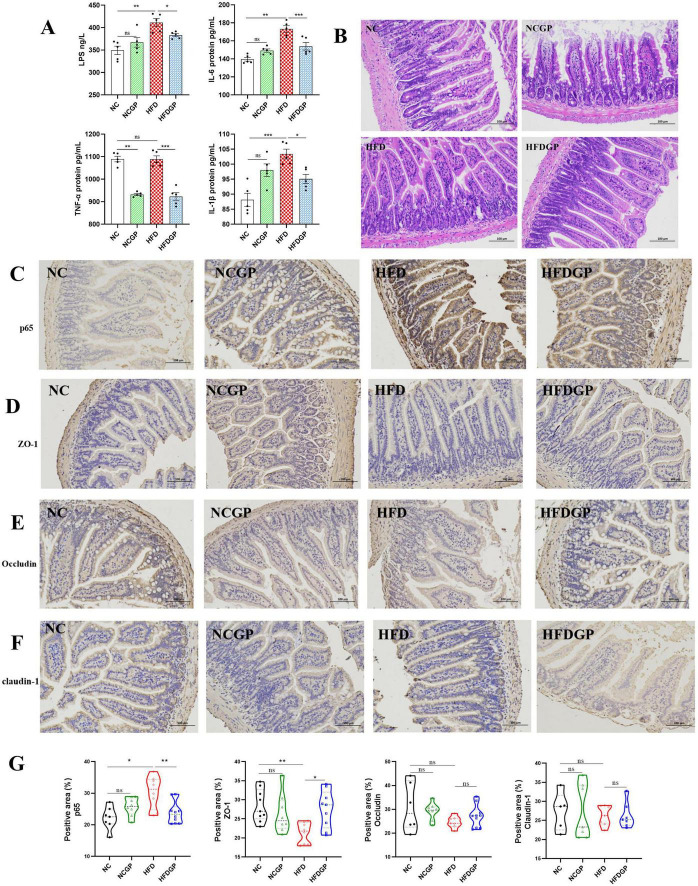
Effect of GAE on HFD-induced systemic inflammation and intestinal barrier dysfunction (*N* = 5). **(A)** the expression levels of inflammatory factors in serum; **(B)** HE staining of ileum; **(C–F)** IHC of p65, Claudin-1, Occludin, ZO-1 in ilea; **(G)** quantitative of IHC staining results. NC: normal chow mice; NCGP: normal chow mice plus GAE; HFD: high-fat diet mice; HFDGP: high-fat diet mice plus GAE. *P < 0.05; **P < 0.01; ***P < 0.001; and ns *P* > 0.05.

As shown in Figure 5A, Group HFD exhibited elevated inflammatory factors (TNF-α, IL-6, IL-1β) and serum LPS levels compared to NC controls, suggesting HFD-induced systemic inflammation. GAE supplementation (Group HFDGP) significantly attenuated these elevations. The HE staining results showed that the structure of the ileum tissue in the NC and NCGP group was clear and the intestinal intestinal structure was intact (Figure 5B). In Group HFD, the ileal villi exhibited shortened and fragmented structures, along with crypt hyperplasia and inflammatory cell infiltration. Compared with Group HFD, Group HFDGP exhibited partial restoration of the villus structure and a significant reduction in inflammatory cell infiltration. Figures 5C–G showed the IHC results of ileum tissue. Compared with Group NC, Group HFD exhibited significantly increased p65 expression. In contrast to Group HFD, the expression of p65 was significantly reduced in Group HFDGP. Group HFD showed reduced expression of tight junction proteins (ZO-1, Occludin, and Claudin-1) compared to Group NC. Although Group HFDGP demonstrated increased expression of all three proteins relative to Group HFD, only ZO-1 showed a statistically significant improvement.

### 3.4 The joint analysis results of 16S rRNA sequencing and untargeted metabolomics

The gut microbiota, serving as a pivotal regulator of host metabolism, actively contributes to the pathological processes of obesity and related metabolic disorders, such as insulin resistance and non-alcoholic fatty liver disease, through the production of bioactive molecules including short-chain fatty acids and secondary bile acids. The combined application of 16S rRNA sequencing and metabolomic profiling enables systematic investigation of tripartite interactions between microbial community structure, metabolic signatures, and host physiological states, providing crucial insights into the “microbiota-metabolism axis” in energy homeostasis dysregulation.

**FIGURE 6:**
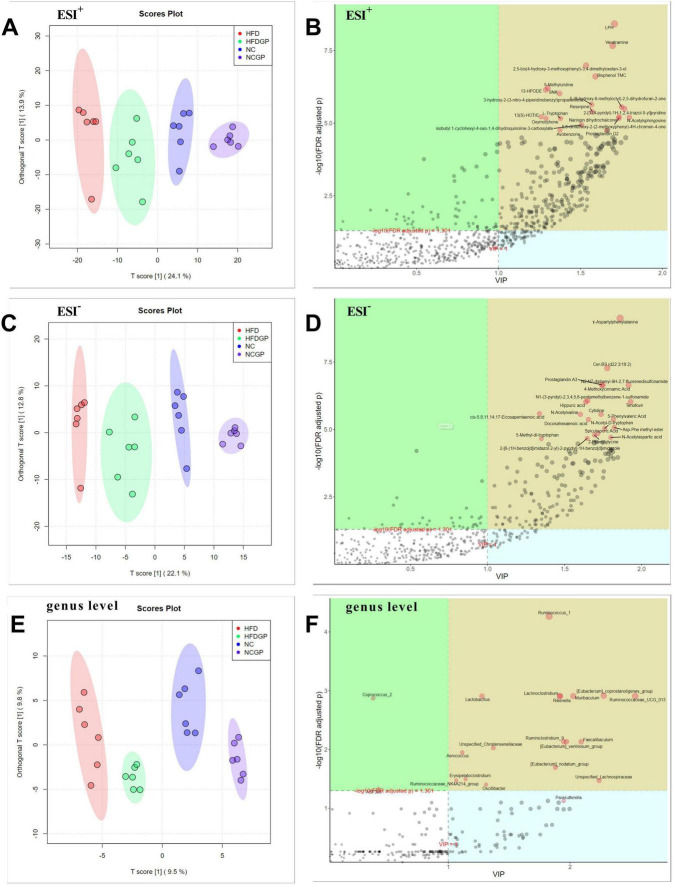
The OPLS-DA score and features Plot of joint analysis. **(A)** OPLS-DA score plot of metabolomics in ESI^+^; **(B)** OPLS-DA features plot of metabolomics in ESI^+^; **(C)**: OPLS-DA score plot of metabolomics in ESI^–^; **(D)** OPLS-DA features plot of metabolomics in ESI^–^; **(E)** OPLS-DA score plot of 16S rRNA sequencing at genus level; **(F)** OPLS-DA features plot of 16S rRNA sequencing at genus level. NC: normal chow mice; NCGP: normal chow mice plus GAE; HFD: high-fat diet mice; HFDGP: high-fat diet mice plus GAE. ESI^+^: electrospray ionization positive mode; ESI^–^: electrospray ionization negative mode.

As depicted in Figure 6, OPLS-DA analysis demonstrated clear separation among the four experimental groups in both metabolic fingerprints (ESI^+/–^ modes) and gut microbiota composition (genus level), confirming substantial diet-induced alterations in microbial ecology and metabolic pathways (Groups HFD vs. NC). Under the ESI^+^ mode, LPH, veratramine, bisphenol TMC, 5-methyluridine, L-tryptophan, and reserpine as key differential metabolites, while ESI^–^ mode revealed γ-aspartylphenylalanine, ceramide Cer-BS (d22:3/18:2), prostaglandin A3, 4-methoxycinnamic acid, and hippuric acid as prominent discriminators. Microbial contributors to group separation included *Ruminococcus*_1, *Muribaculum*, *Ruminococcaceae*_UCG_013, *Rikenella*, *Lachnoclostridium*, *Lactobacillus*, *Faecalibaculum*, Unspecified_*Christensenellaceae*, *Ruminiclostridium*_9, Unspecified_*Christensenellaceae*, *Erysipelatoclostridium*, *Oscillibacter*, *Aerococcus*, and *Parasutterella*.

**FIGURE 7:**
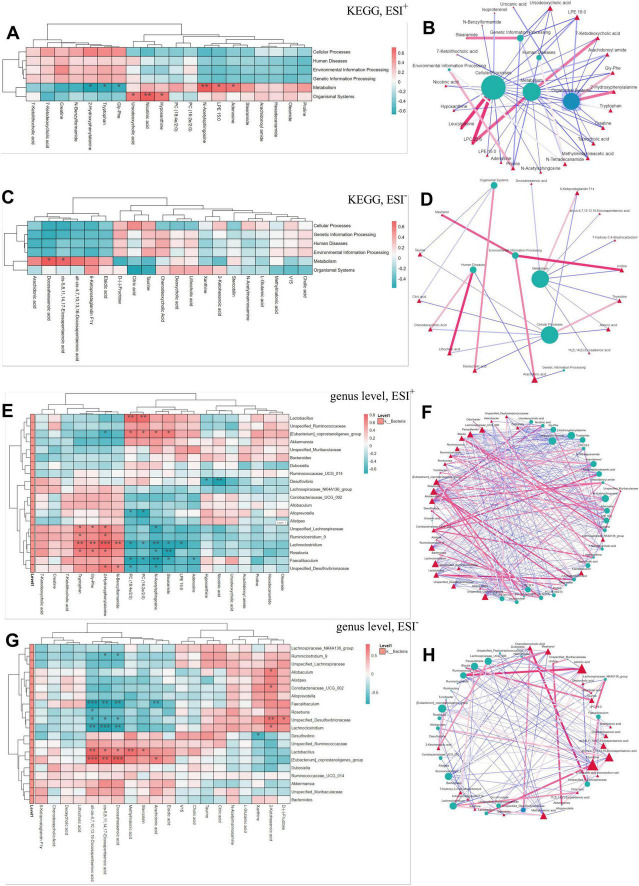
Correlation heatmap and correlation network diagram of joint analysis. **(A)** Correlation heatmap of functional annotations and metabolism in ESI^+^; **(B)** Correlation network diagram of functional annotations and metabolism in ESI^+^; **(C)** Correlation heatmap of functional annotations and metabolism in ESI^–^; **(D)** Correlation network diagram of functional annotations and metabolism in ESI^–^; **(E)** Correlation heatmap of species annotation at genus level and metabolism in ESI^+^; **(F)** Correlation network diagram of species annotation at genus level and metabolism in ESI^+^; **(G)** Correlation heatmap of species annotation at genus level and metabolism in ESI^–^; **(H)** Correlation network diagram of species annotation at genus level and metabolism in ESI^–^. NC, normal chow mice; NCGP, normal chow mice plus GAE; HFD, high-fat diet mice; HFDGP, high-fat diet mice plus GAE. ESI^+^, electrospray ionization positive mode; ESI^–^, electrospray ionization negative mode. *P < 0.05; **P < 0.01; ***P < 0.001.

As presented in Figure 7, correlation analyses revealed distinct metabolic associations. In ESI^+^ mode, N-Acetylmannosamine, LPE15:0, and adenosine showed positive metabolic correlations, while negative associations were observed for 2-hydroxyphenylalanine, tryptophan, and gly-phe. Ursodeoxycholic acid, nicotinic acid, and hypoxanthine demonstrated positive associations with the organismal system (Figure 7A). As depicted in Figure 7B, leucylproline and LPC22:6 as cellular process promoters, contrasting with hypoxanthine, isoproterenol, arachidonoyl amide, adenosine, and LPE18:0 as inhibitors. LPC22:6 and 7-ketodeoxycholic acid exhibited metabolic enhancement properties, opposed by methylimidazoleacetic acid, 7-ketolithocholic acid, arachidonoyl amide, taurocholic acid, and gly-phe. Stearamide positively influenced genetic information processing, while ursodeoxycholic acid, N-tetradecanamide and proline had inhibitory effects. As shown in Figure 7C, Docosahexaenoic acid and cis-5,8,11,14,17-Eicosapentaenoic acid exhibit a significant positive correlation with metabolism. Figure 7D identified 6-ketoprostaglandin F1γ as a metabolic promoter, in contrast to the inhibitory effects of 7-hydroxy-3,4-dihydrocarbostyril, uridine, thymidine, adrenic acid, 11(Z),14(Z)-eicosadienoic acid, and docosahexaenoic acid. Microbial-metabolite interactions analysis demonstrated: *Lactobacillus* positively associated with PC (18:4e/2:0) and PC (16:2e/2:0) (Figure 7E). [*Eubacterium*] *coprostanoligenes*_group correlated positively with PC (18:4e/2:0), PC (16:2e/2:0), N-acetylsphingosine, and stearamide, but inversely with 2-hydroxyphenylalanine. Unspecified *Lachnospiraceae* and *Roseburia* showed positive correlations with tryptophan, gly-phe, and 2-hydroxyphenylalanine, contrasting with negative associations for N-acetylsphingosine and stearamide. *Faecalibaculum* exhibited inverse relationships with PC (18:4e/2:0), PC (16:2e/2:0), N-acetylsphingosine, stearamide, and adenosine (Figure 7F). As demonstrated in Figure 7G, *Ruminiclostridium*_9 was inversely associated with cis-5,8,11,14,17-eicosapentaenoic acid and docosahexaenoic acid. *Faecalibaculum*, *Roseburia*, and *Lachnoclostridium* displayed negative associations with polyunsaturated fatty acids, in contrast to positive correlations observed for [*Eubacterium*] *coprostanoligenes*_group and *Lactobacillus*. Figure 7H demonstrated stercobilin’s negative associations with *Lachnoclostridium*, *Ruminiclostridium*, *Lactobacillus* and unspecified_*Desulfovibrionaceae*. Taurine exhibited positive correlations with Odoribacter and unspecified_*Peptostreptococcaceae*, but showed negative links to multiple genera.

**FIGURE 8:**
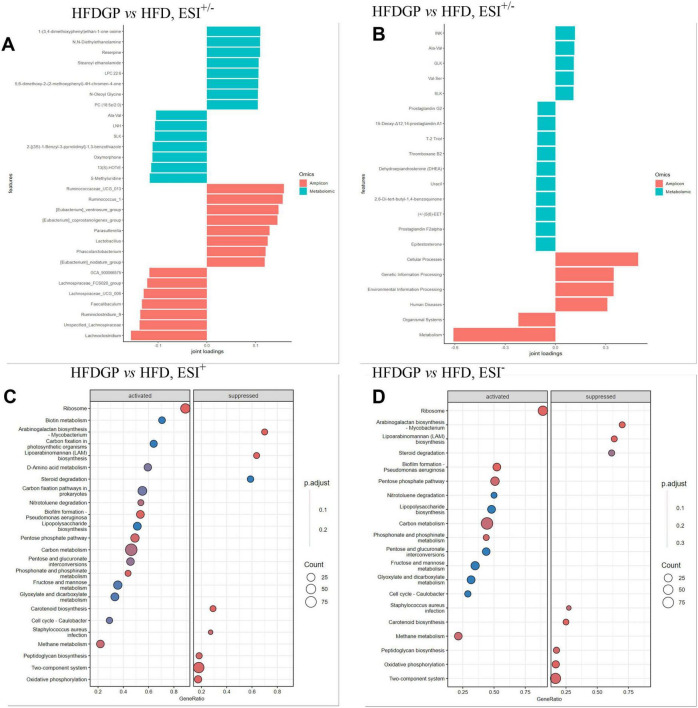
Load value ranking and GESA analysis of joint analysis. **(A)** Load value ranking of species annotation (genus level) and metabolism (ESI^+/–^); **(B)** Load value ranking of functional annotations and metabolism (ESI^+/–^); **(C)** GESA analysis, ESI^+^, HFD vs. HFDGP; **(D)** GESA analysis, ESI^–^, HFD vs. HFDGP. HFD: high-fat diet mice, used as the denominator when calculating Fold Change; HFDGP: high-fat diet mice plus GAE, used as the numerator when calculating Fold Change. ESI^+^, electrospray ionization positive mode; ESI^–^, electrospray ionization negative mode; GESA, gene set enrichment analysis.

According to Figure 8A, Group HFDGP and HFD revealed distinct microbial-metabolic signatures. Group HFDGP showed enrichment of beneficial taxa (*Phascolarctobacterium*, *Lactobacillus*, [*Eubacterium*]_*ventriosum*_group) alongside lipid-regulatory metabolites (stearoyl ethanolamide, LPC22:6, N-oleoyl glycine). Conversely, Group HFD exhibited pro-inflammatory signatures (*Ruminococcaceae*_UCG_013, *Lachnospiraceae*_UCG_006) and stress-related metabolites (oxymorphone, 13(S)-HOTγE). As shown in Figure 8B, functional annotation revealed that biomarkers (GLK and Val-Ser) of Group HFDGP were associated with cellular processes and genetic information processing, whereas markers (prostaglandin G2 and thromboxane B2) of Group HFD were linked to metabolic dysregulation (23). From Figures 8C,D, gene set enrichment analysis (GSEA) revealed that feeding GAE under HFD activated energy metabolism pathways, including the pentose phosphate pathway and ribosome biogenesis. Simultaneously, it suppressed pathogenic pathways such as Staphylococcus aureus infection, Arabinogalactan biosynthesis-Mycobacterium and peptidoglycan biosynthesis.

These findings collectively indicate that GAE intervention modulates gut microbial composition and metabolic output toward anti-inflammatory profiles and lipid homeostasis regulation, contrasting with HFD-induced pro-inflammatory signatures and metabolic disruption.

### 3.5 GAE-regulated gut microbiota alleviates HFD-induced obesity

Changes in intestinal microbiota composition affect host physiology by altering metabolism and immunity (24). Alterations in gut microbiome composition are influenced by diet, host genotype, and the presence of intestinal and metabolic diseases (25). Through FMT experiment, we demonstrated that GAE exerts anti-inflammatory and anti-obesity effects by modulating the gut microbiota.

**FIGURE 9:**
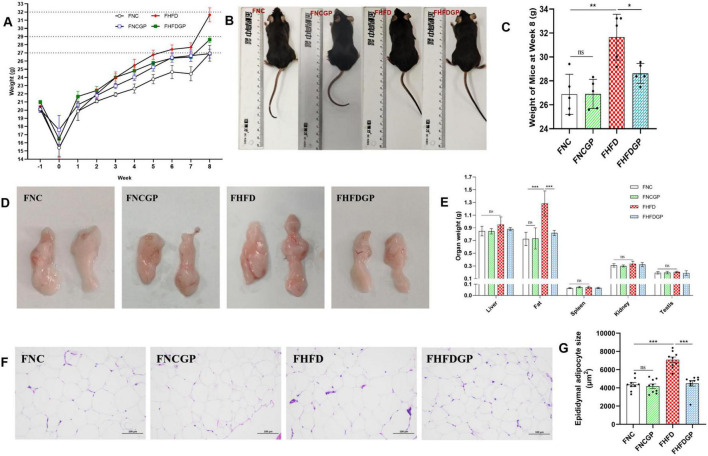
The effects of FMT on the body weight and epididymal fat (*N* = 5). **(A)** Body weight of FMT mice; **(B)** body size of FMT mice; **(C)** body weight of FMT mice in 12th week; **(D)** morphological observations of epididymal adipose tissue; **(E)** weights of different organs; **(F)** HE staining of epididymal adipose; **(G)** mean adipocyte size of epididymal adipose tissue sections. FNC, high-fat diet mice subjected to FMT from NC donors; FNCGP, high-fat diet mice subjected to FMT from NCGP donors; FHFD, high-fat diet mice subjected to FMT from HFD donors; FHFDGP, high-fat diet mice subjected to FMT from HFDGP donors; FMT, fecal microbiota transplantation. *P < 0.05; **P < 0.01; ***P < 0.001; and ns *P* > 0.05.

**FIGURE 10:**
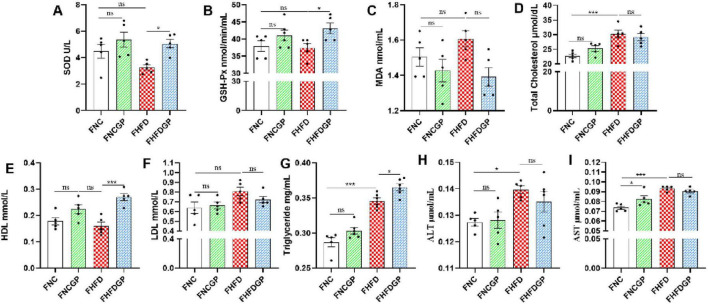
Effect of FMT on oxidative stress and obesity-related biochemical indicators (*N* = 5). **(A)** the superoxide dismutase (SOD) levels in serum; **(B)** the glutathione peroxidase (GSH-Px) levels; **(C)** the malondialdehyde (MDA) levels in serum; **(D)** the total cholesterol levels in serum; **(E)** the high-density lipoprotein (HDL) levels in serum; **(F)** low-density lipoprotein (LDL) levels in serum; **(G)** the triglyceride levels in serum; **(H)** the alanine aminotransferase (ALT) levels in serum; **(I)** the aspartate aminotransferase (AST) levels in serum. FNC: high-fat diet mice subjected to FMT from NC donors; FNCGP: high-fat diet mice subjected to FMT from NCGP donors; FHFD: high-fat diet mice subjected to FMT from HFD donors; FHFDGP, high-fat diet mice subjected to FMT from HFDGP donors. FMT, fecal microbiota transplantation; HDL, high-density lipoprotein; LDL, low-density lipoprotein; ALT, alanine aminotransferase; AST, aspartate aminotransferase. *P < 0.05; ***P < 0.001; and ns *P* > 0.05.

**FIGURE 11:**
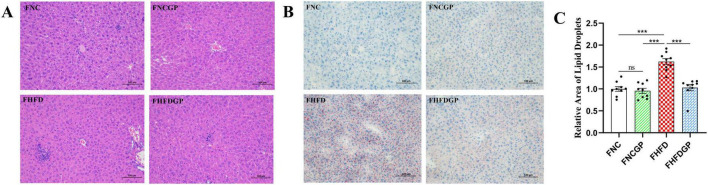
HE staining and Oil Red staining of livers in FMT mice (*N* = 5). **(A)** HE staining of liver; **(B)** Oil Red staining of liver; **(C)** quantitative of lipid droplet area of Oil Red staining. FNC, high-fat diet mice subjected to FMT from NC donors; FNCGP, high-fat diet mice subjected to FMT from NCGP donors; FHFD, high-fat diet mice subjected to FMT from HFD donors; FHFDGP, high-fat diet mice subjected to FMT from HFDGP donors. FMT, fecal microbiota transplantation. ***P < 0.001; and ns *P* > 0.05.

**FIGURE 12:**
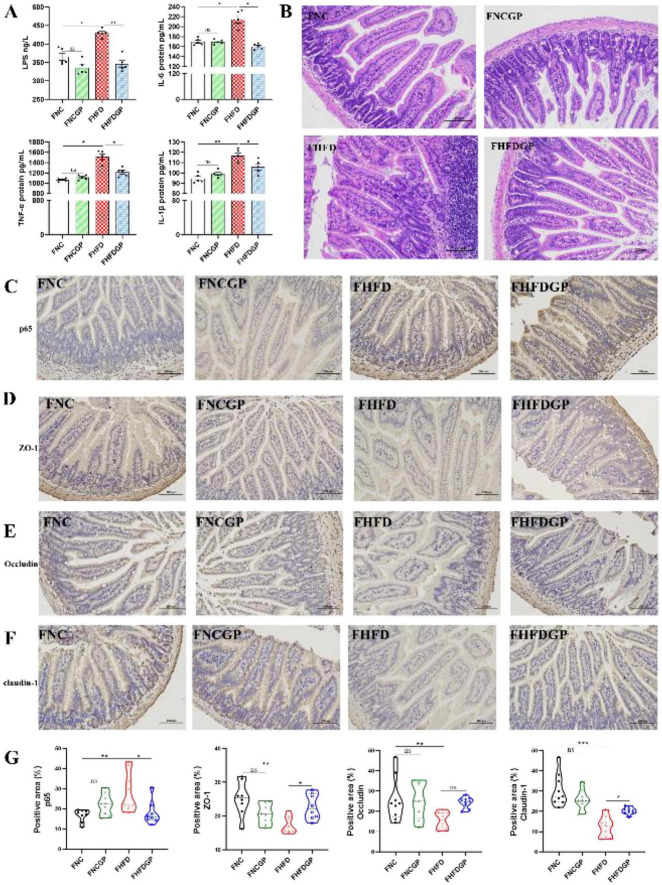
Effect of FMT on HFD-induced inflammation and gut epithelial impairment (*N* = 5). **(A)** The expression levels of inflammatory factors in serum; **(B)** HE staining of ileum; **(C–F)** IHC of p65, Claudin-1, Occludin, ZO-1 in ilea; **(G)** quantitative of IHC staining results. FNC, high-fat diet mice subjected to FMT from NC donors; FNCGP: high-fat diet mice subjected to FMT from NCGP donors; FHFD, high-fat diet mice subjected to FMT from HFD donors; FHFDGP, high-fat diet mice subjected to FMT from HFDGP donors. FMT, fecal microbiota transplantation. *P < 0.05; **P < 0.01; ***P < 0.001; and ns *P* > 0.05.

As shown in Figure 9, during the first week when mice were treated with high doses of antibiotics, their body weight significantly decreased. By the 8th week of FMT, The body weight, epididymal fat weight and adipocyte size of mice in Group FNC and FHFDGP were all significantly lower than those in Group FHFD. Although there was no statistically significant difference in MDA levels, across the groups, Group FHFDGP showed significantly elevated SOD and GSH-Px activity relative to the FHFD group (Figures 10A–C). The levels of total cholesterol, triglyceride, ALT and AST in Group FNC were significantly lower than those in Group FHFD, and the levels of triglyceride and insulin in Group FHFDGP were significantly lower than those in Group FHFD (Figures 10D–I). Transplantation of gut microbiota from both Group NC and HFDGP was found to be effective in alleviating obesity induced by HFD. It is worth noting that the gut microbiota in the two groups might exert their effects through different biological pathways. According to Figure 11, Group FHFD exhibited the largest lipid droplet area in liver tissue and a small amount of inflammatory cell infiltration. The lipid droplet areas in the liver tissues of Group FNC, FNCGP, and FHFDGP were all significantly smaller than that of Group FHFD. The findings presented in Figure 12 reveal that both Group FNC and FHFDGP exhibited significantly lower concentration levels of inflammatory factors (TNF-α, IL-6, IL-1β) and serum LPS compared to Group FHFD (*P* < 0.05). Both Group FNC and FNCGP showed intact villous structures in the ileum, with abundant goblet cells and few inflammatory cell infiltrations. In Group FHFD, the ileal villi were atrophied, the crypt structure was disrupted, and there was significant inflammatory cell infiltration. Visually, inflammatory cell infiltration was significantly less in Group FHFDGP than in Group FHFD. The ileum tissue in Group FHFD exhibited significantly higher positive expression of p65 compared to Groups FNC and FHFDGP. Group FHFDGP exhibited significantly higher expression levels of the tight junction proteins claudin-1 and ZO-1 compared to Group FHFD. In summary, during the high-fat diet intervention period, the gut microbiota regulated by feeding GAE exhibited a similar effect of reducing obesity as the healthy gut microbiota community.

## 4 Discussion

In this study, we systematically evaluated the anti-obesity effects of GAE and deciphered its underlying mechanisms. Contrary to the traditional view of simply supplementing antioxidants, our findings demonstrate that GAE functions as a multi-target modulator that alleviates HFD-induced obesity, hepatic steatosis, and systemic inflammation primarily by reshaping the gut microbiota and reprogramming metabolic profiles. Crucially, through FMT, we established a causal link between GAE-modulated gut dysbiosis and the amelioration of metabolic phenotypes, providing robust evidence for the therapeutic potential of GAE via the “gut-metabolism-immune” axis.

As shown in Table 1, GAE contains a large number of bioactive components that can improve oxidative stress and lipid metabolism. For instance, carnosic acid, quercetin, kaempferol and acid have been proven to possess antioxidant and anti-inflammatory properties (26–29). These active ingredients of GAE can alleviate systemic oxidative stress and enhance the overall metabolic status of the body by increasing the enzymatic activities of SOD and GSH-Px, as well as decreasing the levels of MDA (Figure 1). Adipocyte hypertrophy is a hallmark feature of obesity in adulthood (30). Hypertrophied adipocytes contribute to lipid metabolic disorders, enhance fatty acid release, and consequently exacerbate insulin resistance (31, 32). Moreover, obesity induces increased inflammatory responses within adipose tissue, where chronic low-grade inflammation further intensifies insulin resistance (33). Elevated levels of cholesterol and triglycerides represent another metabolic abnormality associated with obesity (34). The accumulation of these lipids is not only linked to adipocyte hypertrophy but also strongly correlated with alterations in lipid metabolism within adipose tissue (35). Additionally, hepatic lipid deposition, a common complication of obesity, is closely tied to the pathological progression of adipose tissue dysfunction (36). Our research findings have demonstrated that GAE not only effectively suppressed HFD-induced weight gain and adipocyte hypertrophy but also mitigated metabolic disorders by improving lipid homeostasis, reducing hepatic lipid accumulation, enhancing insulin sensitivity, and promoting glucose tolerance (Figures 2–4). Besides, obesity is closely linked to low-grade inflammation, compromised intestinal barrier function, and gut microbiota dysbiosis. Current evidence indicates that the intestinal expression levels of tight junction proteins are significantly downregulated in obese individuals, which consequently enhances intestinal barrier permeability (37). This compromised barrier function promotes the systemic dissemination of inflammatory mediators through circulatory transport, ultimately precipitating a cascade of systemic inflammatory responses (38).

Obesity correlates with dysfunction in the intestinal barrier and systemic inflammation (39, 40). Our results showed that GAE significantly upregulated ZO-1, Occludin, and Claudin-1 expression and inhibited the NF-κB signaling pathway in the intestine (Figure 5). This reinforcement of the gut barrier likely blocks the entry of pathogen-associated molecular patterns (PAMPs), thereby cutting off the inflammatory cascade at its source. This finding explains the observed reduction in serum LPS and inflammatory cytokines, highlighting gut barrier protection as a key facet of GAE’s anti-obesity efficacy.

The integration of 16S rRNA sequencing and non-targeted metabolomics provided deeper insights into how GAE remodels the gut ecosystem (41). This approach not only highlights structural features of the microbial community and dynamic changes in metabolites but also uncovers functional linkages between specific bacterial genus and critical metabolites. Figure 6 displayed the OPLS-DA analysis results, revealing distinct clustering among the four experimental groups in terms of metabolic fingerprints (ESI^+/–^ mode) and gut microbiota composition at the genus level. These key differential metabolites are implicated in diverse physiological processes and show strong associations with obesity-related metabolic disorders. For instance, L-Tryptophan supplementation has been shown to significantly attenuate weight gain induced by HFD and enhance insulin sensitivity, thereby mitigating key metabolic disturbances associated with obesity (42). Besides, the indole metabolites of L-tryptophan contribute to maintaining intestinal mucosal integrity, strengthening the intestinal barrier function, and exerting anti-inflammatory effects, thereby supporting overall gut health (43). Furthermore, shifts in specific genera including *Ruminococcus*_1 and *Muribaculum* indicated structural remodeling of the gut microbiota community under GAE intervention. These microorganisms play critical roles in carbohydrate, protein, and lipid metabolism, with their abundance alterations potentially affecting host nutrient absorption, energy expenditure, and adipose deposition. Figure 7 revealed the complex interaction network between metabolites and microorganisms. *Lactobacillus* and *Roseburia* were associated with enhanced intestinal barrier function via production of short-chain fatty acids (e.g., butyrate) (44, 45). These genera showed positive correlations with PC (18:4e/2:0), PC (16:2e/2:0) and tryptophan metabolism, suggesting potential mechanisms for lipid homeostasis improvement and anti-adipogenic effects. The (*Eubacterium*) *coprostanoligenes*_group exhibited significant correlations with docosahexaenoic acid and eicosapentaenoic acid, which might mediate anti-inflammatory responses to enhance insulin sensitivity (46). In contrast, *Faecalibaculum* and *Desulfovibrio* showed negative correlations with N-acetylsphingosine and hypoxanthine, respectively. These associations might reflect compromised gut barrier integrity through sphingomyelinase pathway dysregulation or hydrogen sulfide overproduction, exacerbating metabolic inflammation. However, it is important to emphasize that these associations are correlational and do not imply direct causality. They represent a hypothesis-generating framework that necessitates subsequent experimental verification, such as in vitro strain cultivation or co-culture assays, to definitively establish mechanistic links.

Studies have demonstrated that LPC 22:6 exerts anti-inflammatory effects through the inhibition of pro-inflammatory leukotriene and cytokine formation, as well as by promoting the production of lipoxin A (47). Reguero et al. demonstrated that N-oleoyl glycine activated the PPARα pathway, thereby enhancing lipid metabolism in the liver (48). Studies have shown that an increased abundance of *Phascolarctobacterium* is positively associated with weight loss (49, 50). *Lactobacillus*. gasseri BNR17 reduces obesity by blocking the formation of fat cells through PPARγ and *Lactobacillus*. KY1032 reduce obesity by boosting GLP-1 secretion via TGR5 (51, 52). The results of Figure 8 demonstrated that GAE significantly ameliorated HFD-induced gut microbiota dysbiosis and lipid metabolic disorders by enriching lipid-regulating metabolites (LPC 22:6, and N-oleoyl glycine) and beneficial genera (*Phascolarctobacterium* and *Lactobacillus*). In contrast, Group HFD exhibited a predominance of pro-inflammatory taxa (*Ruminococcaceae*_UCG_013) and stress-related metabolites (13(S)-HOTγE), indicative of systemic oxidative stress and inflammatory activation. Functional annotation further revealed that HFD-specific markers (prostaglandin G2 and thromboxane B2) aligned with dysregulated metabolic and organismal systems, particularly pro-inflammatory eicosanoid pathways (23, 53).

To contextualize the efficacy of GAE, we compared our findings with other well-established plant-derived anti-obesity agents. For instance, lycopene is renowned for its multi-faceted anti-obesity properties, which involve modulating lipid metabolism, reducing systemic inflammation, and improving insulin sensitivity (54, 55). Similarly, catechins have been shown to exert anti-obesity effects through interconnected mechanisms, including the suppression of chronic low-grade inflammation, enhancement of lipid oxidation, and promotion of adipocyte browning (56, 57). These mechanisms align with the anti-inflammatory and lipid-regulating effects observed in GAE. However, our study reveals a distinct advantage of GAE: its potent modulation of the “microbiota-metabolite” axis. Unlike lycopene or catechins, whose effects are often attributed directly to host tissue signaling, GAE appears to drive metabolic improvements significantly through the enrichment of specific functional metabolites and beneficial gut bacteria. GSEA highlighted that GAE activated energy metabolism pathways (pentose phosphate pathway and ribosome biogenesis) while suppressing pathogenic pathways (Staphylococcus aureus infection and peptidoglycan biosynthesis). Oxidative phosphorylation serves as a critical process for energy production in mitochondria, and its inhibition results in mitochondrial dysfunction. Notably, the inhibition of oxidative phosphorylation and methane metabolism in HFD mice suggested compromised mitochondrial function and altered gut ecological balance. These findings collectively implicating GAE as a multi-target modulator of the “microbiota-metabolite-immune” axis to counteract HFD-driven metabolic perturbations.

FMT experiment provided definitive evidence for the effect of the gut microbiota. Mice receiving fecal microbiota from GAE-treated donors (Group FHFDGP) exhibited resistance to HFD-induced obesity, mirroring the phenotype of the direct GAE intervention group (Figures 9–12). By the 8th week of FMT, both the FNC and FHFDGP groups showed significantly lower body weight, epididymal fat mass, adipocyte size, and hepatic lipid droplet area compared to Group FHFD, indicating that the gut microbiota modulated by GAE effectively suppressed weight gain and fat accumulation, thereby alleviating obesity-related phenotypes. Metabolically, although no significant differences in oxidative stress levels were observed among groups, Group FHFDGP demonstrated significantly higher activities of SOD and GSH-Px than Group FHFD. Furthermore, serum triglyceride and insulin levels in Group FHFDGP were markedly reduced compared to Group FHFD. In the intestine, Group FHFD displayed villus atrophy and pronounced inflammatory infiltration. Notably, Group FHFDGP showed reduced intestinal inflammation compared to Group FHFD, accompanied by elevated expression of tight junction proteins, suggesting that GAE-modulated gut microbiota restores the compromised intestinal barrier and alleviates inflammatory responses. Collectively, the NC and NCGP recipients showed reduced adiposity, improved insulin sensitivity, and restored intestinal barrier function compared to the HFD recipients. This implies that the altered microbiota itself is sufficient to confer metabolic benefits, independent of the continuous presence of GAE phytochemicals. This “transmissible” protective effect underscores the potential of GAE to serve as a prebiotic agent that cultivates a resilient and metabolically beneficial gut microbiome.

Our results have significant implications for the functional food industry. Given its traditional use as a foodstuff and the robust anti-obesity effects demonstrated here, *Gnaphalium affine* is a prime candidate for development into a variety of functional food products, such as dietary supplements, fortified beverages, or specialized nutritional powders aimed at weight management.

Despite these promising findings, our study has limitations. While we identified potential key active components in GAE (Table 1), we used the whole extract for intervention. Future studies should isolate specific compounds (e.g., specific flavonoids) to pinpoint the exact molecules responsible for the microbiota-modulating effects. Additionally, although we observed changes in bile acids and lipid metabolites, the specific bacterial enzymes involved in these metabolic transformations remain to be characterized using metagenomics or metatranscriptomics. Finally, while the multi-omics analysis identified potential microbe-metabolite interactions, specific validation via in vitro co-culture systems is needed to confirm the direct metabolic output of these bacterial species. While the mouse model provides valuable mechanistic insights, clinical trials are necessary to validate the translational potential of GAE in human obesity management.

## 5 Conclusion

In summary, this study systematically demonstrates that GAE mitigates obesity and metabolic disorders induced by a high-fat diet. The underlying mechanism involves modulation of the gut microbiome, resulting in enrichment of beneficial bacteria, production of beneficial metabolites, restoration of intestinal barrier function, and attenuation of systemic inflammation. The causal role of gut microbiota has been firmly established by fecal microbiome transplantation. These findings provide a solid scientific foundation for the development of *Gnaphalium affine* as a promising functional food ingredient for promoting metabolic health and managing obesity.

## Data Availability

The data presented in this study are publicly available. The data can be found at: https://www.ncbi.nlm.nih.gov/ accession PRJNA947507.

## Footnotes

1
https://www.bioincloud.tech
